# Evaluation of freshwater heavy metals accumulation effect on oxidative stress, Metallothionein biosynthesis and histopathology of *Procambarus clarkii* (Girard,1985) collected from three locations in the Delta region, Egypt

**DOI:** 10.1186/s40850-023-00183-8

**Published:** 2023-09-18

**Authors:** Mahy M. Mona, Mai L. Younis, Aalaa I. Atlam

**Affiliations:** Zoology Department, Faculty of Science, Tanta University, Tanta, 31572 Egypt

**Keywords:** Oxidative stress, Protein profile, *Procambarus clarkii*, Metallothioneins, Delta region, Histopathology

## Abstract

**Background:**

In this study, the effect of heavy metals accumulation influence was evaluated on adult crayfish *Procambarus clarkii* (Decapoda, Astacidea) collected from three different Governmental locations (Kafr El-Shaikh, El-Menofya, and El-Gharbiya) of the Egyptian Delta. The activity of super oxidase dismutase (SOD), catalase (CAT), and glutathione peroxidase (GPX) of gills, hepatopancreas, and muscle tissue were measured. SDS Polyacrylamide gel electrophoresis (SDS-PAGE) and West blotting technique were performed to detect MT Protein expression.

**Results:**

The results revealed that Kafr El-Shaikh reflected the highest Superoxide dismutase (SOD), Catalase, and Glutathione S-transferase (GST) activity levels (97.2 u/100 mg, 28.5 u/100 mg, and 8.3 nmol mg (-1) protein min (-1) respectively. Superior protein polymorphism % (30%) remarked collected Freshwater crayfish *P. clarkii* from Kafr El-Shaikh location. Varied protein polymorphism % was shown between collected crayfish from El-Menofya, and El-Gharbiya locations (5.5 and 6.2 respectively) Increasing Metallothioneins intensity (15.4%) for collected Freshwater crayfish *Procambarus clarkii* from Kafr El-Shaikh Location.

**Conclusion:**

Heavy metal stress influences antioxidant status and also induces increasing Metallothioneins intensity, especially samples that were collected from the Kafr El-Shaikh area.

## Background

The deterioration of the Nile River's water quality is a major issue in Egypt. Until it reaches the Delta, the water quality released from Aswan High Dam (AHD) is relatively clean and displays no degradation [1]. Egypt's main issue is the rise in pollution brought on by the Nile's low water level, particularly after the Ethiopia Dam is completed. Heavy metals are the most prevalent pollutant in the Nile River, with contamination originating from a variety of anthropogenic sources including industrial, agricultural, and home effluents. Heavy metal poisoning of surface water is a serious ecological risk due to its potential toxicity for both humans and the environment. They can bioaccumulate in the food chain since they are not biodegradable [2, 3].

The ability of some animals to accumulate relatively high levels of some contaminants, even from greatly diluted solutions, even without visible impacts, forms the basis for the biomonitoring of pollutants employing accumulator species. When their levels in the natural environment are below the detection limits of the generally employed methods, this methodology enables the measurement of trace element concentrations. Additionally, although the pollutant concentrations in the water only reflect the situation at the time of sampling, the pollutant concentrations in the organism are a product of both past and present pollution levels in the environment in which the organism lives [4].

Crayfish as well as other decapods are known to be vulnerable to pollution in freshwater ecosystems. These organisms are particularly responsive to changes in aquatic environments because of their sensitivity to changes in water quality [5, 6]. Both in the aquatic environment and in the laboratory, crayfish have been utilized as bioindicators. They show a strong preference for collecting contaminants in their tissues [7, 8] and respond to a variety of chemicals [5, 9]. As a result, they have the potential to be used as bioindicators in actual monitoring in industrial applications.

Organisms have developed a multitude of defense systems in response to exposure to free radicals from a variety of sources [10]. Preventive processes, healing mechanisms, physical defenses, and antioxidant defenses are all part of the defense against free radical-induced oxidative stress. Superoxide dismutase (SOD), catalase (CAT), and glutathione peroxidase are examples of enzymes that provide antioxidant protection (GPx). Vitamin C, tocopherol, glutathione (GSH), carotenoids, flavonoids, and other antioxidants are examples of non-enzymatic antioxidants. The antioxidant system in the body regulates the production and clearance of reactive oxygen species (ROS) [11–14].

Superoxide dismutases (SODs) are enzymes produced by organisms that can survive in the presence of oxygen. They help superoxide be transformed into oxygen and hydrogen peroxide through catalysis. SOD enzymes regulate a variety of reactive oxygen species (ROS) and reactive nitrogen species levels through their activity, which limits the potential toxicity of these molecules while also regulating a wide range of cellular processes that are governed by their signaling functions [15]. Furthermore, CAT can efficiently catalyze the breakdown of hydrogen peroxide (H2O2) to maintain the proper ratio between de novo H2O2 synthesis and rapid clearance, which is crucial for innate immunity. Furthermore, as H2O2 is involved in the sensing and signaling of various biological events, the up- or down-regulation of catalase can affect a variety of biological processes, such as growth, differentiation, emigration, and apoptotic [16]. In addition, glutathione S-transferases (GSTs) can reduce lipid hydroperoxides through their Se-independent glutathione-peroxidase activity [17].

Metallothioneins (MTs) are non-enzymatic, low-molecular-weight proteins rich in cysteine, free of aromatic amino acids, and heat stable. Metal stress in invertebrates could be detected using MTs as a biomarker. On the basis of molecular weights, UV absorption spectra, isoelectric points, and amino acid contents, crustacean MTs were shown to be very comparable to vertebrate MTs [18]. The biological activities of MTs are as follows: first, these proteins provide a non-toxic zinc and copper reserve for the creation of metalloenzymes, allowing several cellular processes to maintain homeostasis. Second, MTs can inhibit non-essential metals (Cd, Hg, or Ag) from binding within cells, hence limiting their harmful potential [19].

The current study aimed to evaluate the effect of heavy metals on some tissues of crayfish *P. clarkia* (as a bioindicator), from three separate government locations (Kafr El-Shaikh, El-Menofya, and El-Gharbiya) of the Egyptian Delta, focusing on the immune and histological response of collected samples.

## Results

**Fig. 1:**
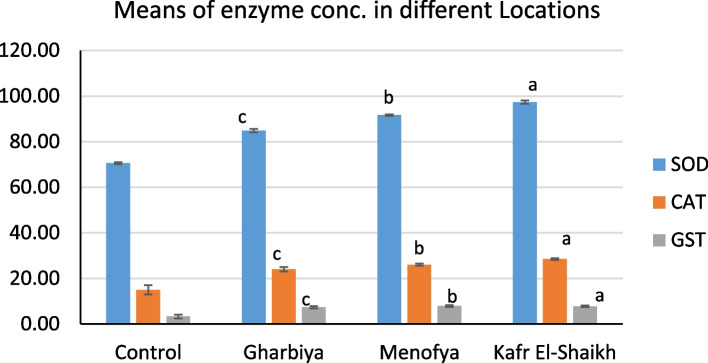
Collected Freshwater crayfish *Procambarus clarkii* form Control (1), El-Gharbia (2), Kafr El-Sheikh (3) and El-Menofia (4) locations for three oxidative stress enzymes

Oxidative stress markers including Superoxide dismutase (SOD), Catalase, and Glutathione S-transferase (GST) are estimated for collected Freshwater crayfish *Procambarus clarkii* from three locations as shown in Fig. 5 (Kafr El-Shaikh, El-Menofya, and El-Gharbiya) comparing with the reference sample. As shown in Table 1 and Fig. 1, among three locations, Kafr El-Shaikh reflected the highest Significant levels (*P* < 0.001) of Superoxide dismutase (SOD), Catalase, and Glutathione S-transferase (GST) activity (97.2 u/100 mg, 28.5 u/100 mg and 8.3 nmol/mg protein min (^−1^) respectively. On the contrary, the lowest Oxidative stress markers values were remarked taken crap from El-Gharbiya (85.5 u/100 mg, 24 u/100 mg, and 7.4 nmol /mg protein min (^−1^) respectively, compared to the control and other locations On the other hand, the lowest level of enzymes were recorded in El-Gharbiya, compared to other locations, but still significsntly higher than the control group readings.

**Table 1:** Average of Superoxide dismutase (SOD), Catalase (CAT) and Glutathione S-transferase (GST) for collected Freshwater crayfish* P. clarkii* tissue from El-Gharbiya, Kafr El-Shaikh, and El-Menofya locations

Location enzyme	Control	El-Gharbiya	El-Menofya	Kafr El-Shaikh
SOD	70.6 ± 0.265	84.833 ± 0.441 ^c^	91.667 ± 0.426 ^b^	97.367 ± 0.203 ^a^
CAT	15 ± 1.155	24 ± 0.577 ^c^	26.067 ± 0.233 ^b^	28.5 ± 0.289 ^a^
GST	3.3 ± 0.458	7.333 ± 0.285 ^c^	7.733 ± 0.2028 ^b^	7.9 ± 0.208 ^a^

^a^Means highest significant

^b^Means significant difference number 2

^c^Means the lowest significant

**Fig. 2:**
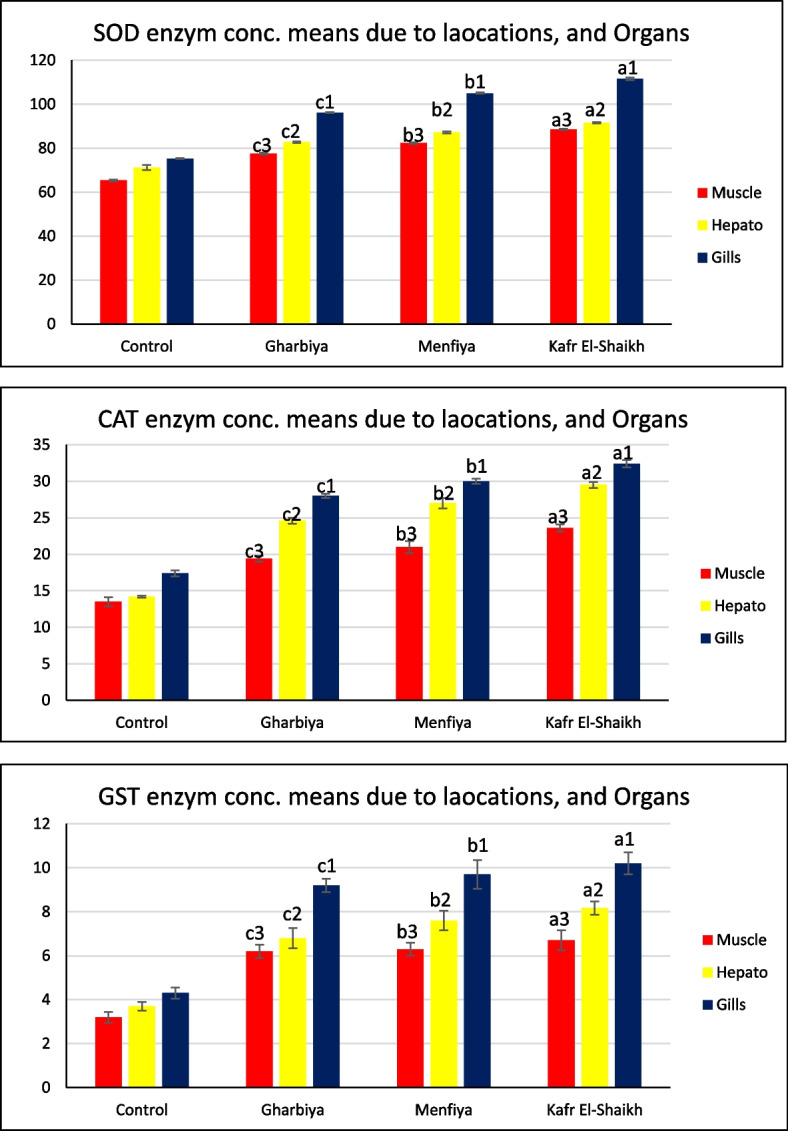
Superoxide dismutase (SOD), Catalase (CAT), and Glutathione S-transferase (GST) levels of collected *Freshwater crayfish Procambarus clarkii* Muscle, hepatopancreas and Gills from El-Gharbia, Kafr El-Sheikh and Menofia locations

Different organs (muscle, hepatopancreas, and gills) of collected Freshwater crayfish* P. clarkii* from three locations reflected varied responses against heavy metals stresses. Generally, gills recorded the highest readings among all organs, and showed the highest significant level (*P* < 0.001) of Superoxide dismutase (SOD), Catalase, and Glutathione S-transferase (GST) content in all of the studied areas, compared with control gills as shown in Table 2 and Fig. 2.

**Table 2:** Superoxide dismutase (SOD), Catalase (CAT), and Glutathione S-transferase (GST) for collected *Freshwater crayfish P. clarkii* Muscle, hepatopancreas, and Gills from El-Gharbiya, Kafr El-Shaikh and El-Menofya locations

Enzyme	Organ Location	Muscle	Hepato	Gills
SOD	Control	65.3 ± 0.208	71.233 ± 0.677	75.3 ± 0.115
	Gharbiya	77.5 ± 0.231 ^c3^	82.767 ± 0.203 ^c2^	96.233 ± 0.145 ^c1^
	Menfiya	82.333 ± 0.203 ^b3^	87.2 ± 0.231 ^b2^	105.3 ± 0.173 ^b1^
	Kafr El-Shaikh	88.533 ± 0.176 ^a3^	91.567 ± 0.203 ^a2^	111.567 ± 0.376 ^a1^
CAT	Control	13.533 ± 0.348	14.167 ± 0.088	17.4 ± 0.231
	Gharbiya	19.4 ± 0.231 ^c3^	24.6 ± 0.231 ^c2^	28 ± 0.173 ^c1^
	Menfiya	21.467 ± 0.47 ^b3^	26.6 ± 0.405 ^b2^	30.133 ± 0.203 ^b1^
	Kafr El-Shaikh	23.567 ± 0.291 ^a3^	29.533 ± 0.233 ^a2^	32.433 ± 0.291 ^a1^
GST	Control	3.233 ± 0.145	3.7 ± 0.115	4.267 ± 0.145
	Gharbiya	6.167 ± 0.176 ^c3^	6.8 ± 0.265 ^c2^	9.233 ± 0.176 ^c1^
	Menfiya	6.3 ± 0.173 ^b3^	7.567 ± 0.26 ^b2^	9.667 0.376 ^b1^
	Kafr El-Shaikh	6.7 ± 0.265 ^a3^	8.167 ± 0.176 ^a2^	10.2 ± 0.289 ^a1^

^a^Means highest significant, take the forms (a1, a2 a3) for organs (Gills, Hepato, and Muscle) respectively

^b^Means significant difference number 2, take the forms (b1, b2 b3) for organs (Gills, Hepato, and Muscle) respectively

^c^Means the lowest significant, take the forms (c1, c2 c3) for organs (Gills, Hepato, and Muscle) respectively

Lowest enzyme readings were observed in muscles samples from all studied areas, compared to other organs. Also, lowest muscle enzymes were found in Gharbiya, but on the other hand, still significantly higher than the control readings.

Also, it was found that all organs from Kafr El-Shaikh samples’ showed the highest readings compared to organs from other locations and control group, which emphasize results mentioned in the previous table.

**Fig. 3:**
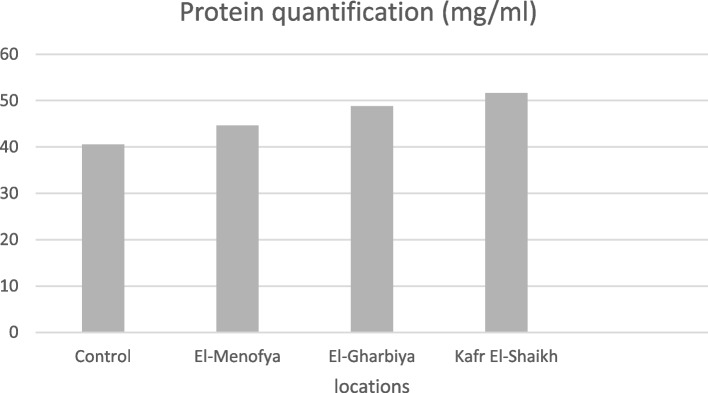
Total protein value of control and collected *Freshwater crayfish Procambarus clarkii* from El-Menofia, El-Gharbia and Kafr El-Sheikh locations

Quantification and fragmentation of total soluble proteins for collected Freshwater crayfish *P. clarkii* from three locations (Kafr El-Shaikh, El-Menofya, and El-Gharbiya) were performed, and a positive correlation was detected between heavy metal stresses and superior protein content (Table 3 and Fig. 3).

**Table 3:** Total protein value of control and collected Freshwater crayfish* P. clarkii* from El-Menofya, El-Gharbiya, and Kafr El-Shaikh locations

	Locations			
	Control	El-Menofya	El-Gharbiya	Kafr El-Shaikh
Protein quantification (mg/ml)	40.56	44.64	48.81	51.63

Collected Freshwater crayfish *P. clarkii* from Kafr El-Shaikh,which was remarked with the highest heavy metals content, showed highly distinguishable protein content (51.63 mg/ml) compared with the control sample (40.56 mg/ml), and compared to other locations. Moderate and low protein content for collected samples from El-Gharbiya and El-Menofya,(48.81 and 44.64 and mg/ml) indicated our hypothesis.

**Fig. 4:**
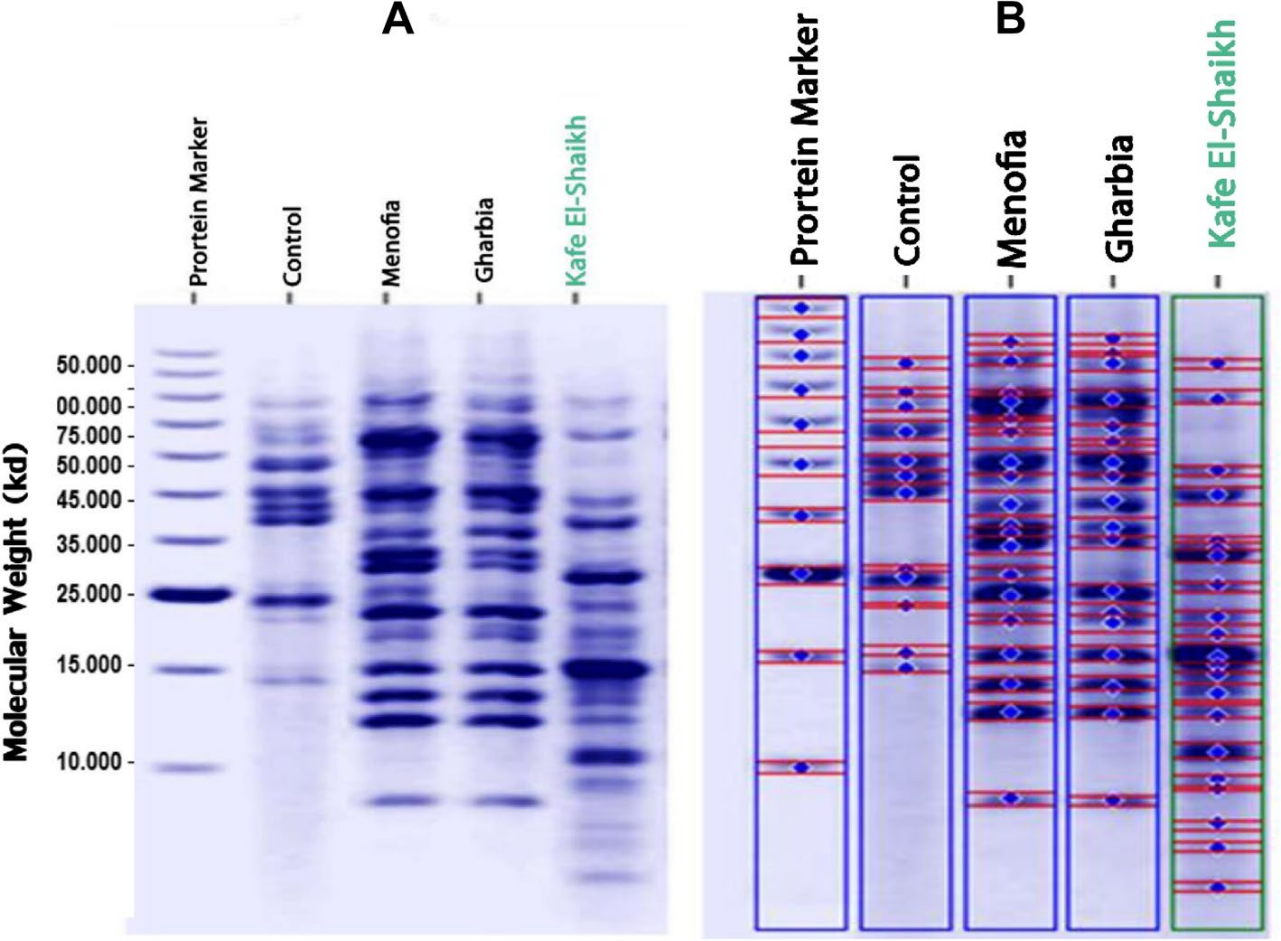
Electrophoretic patterns (**A**) and computerized detection (**B**) for control and collected *Freshwater crayfish Procambarus clarkii* from El-Menofia, El-Gharbia, and Kafr El-Sheikh locations

Protein electrophoretic patterns for collected Freshwater crayfish *P. clarkii* from the three locations were studied to evaluate the heavy metal effect. As shown in Fig. 4 and Table 4, collected samples from Kafr El-Shaikh location reflected superior protein band number (20 bands). On the other hand, the lowest protein bands were observed in depurated Freshwater crayfish *P. clarkii* (12 bands).

**Table 4:** Total, Polymorphic, Monomorphic bands and Polymorphism % for control and collected *Freshwater crayfish P. clarkii* from El-Menofya, El-Gharbiya, and Kafr El-Shaikh locations

	**Control**	**Locations**		
		**El-Menofya**	**El-Gharbiya**	**Kafr El-Shaikh**
**Total** **bands**	12	18	16	20
**Polymorphic** **Bands**	0	1	1	6
**Monomorphic** **Bands**	12	17	15	14
**Polymorphism %**	0	5.5	6.2	**30**

**Fig. 5:**
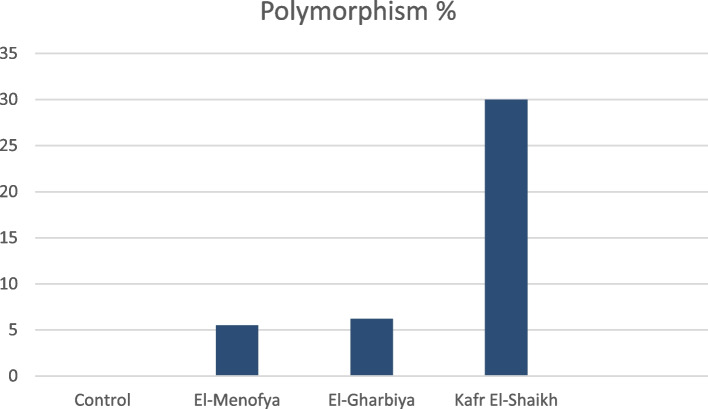
Polymorphism % for control and collected *Freshwater crayfish Procambarus clarkii* from Menofia, Gharbia, and Kafr El-Sheikh locations

As a direct influence of heavy metals stress, a positive relationship was detected between protein polymorphism % and degree of exposure to heavy metals (Table 4 and Fig. 5). Superior protein polymorphism % (30%) remarked from Kafr El-Shaikh’s samples. Also, varied protein polymorphism % between collected samples from El-Menofya, and El-Gharbiya locations (5.5% and 6.2% respectively) was shown. Furthermore, control samples reflected 100% of genetic similarity.

**Fig. 6:**
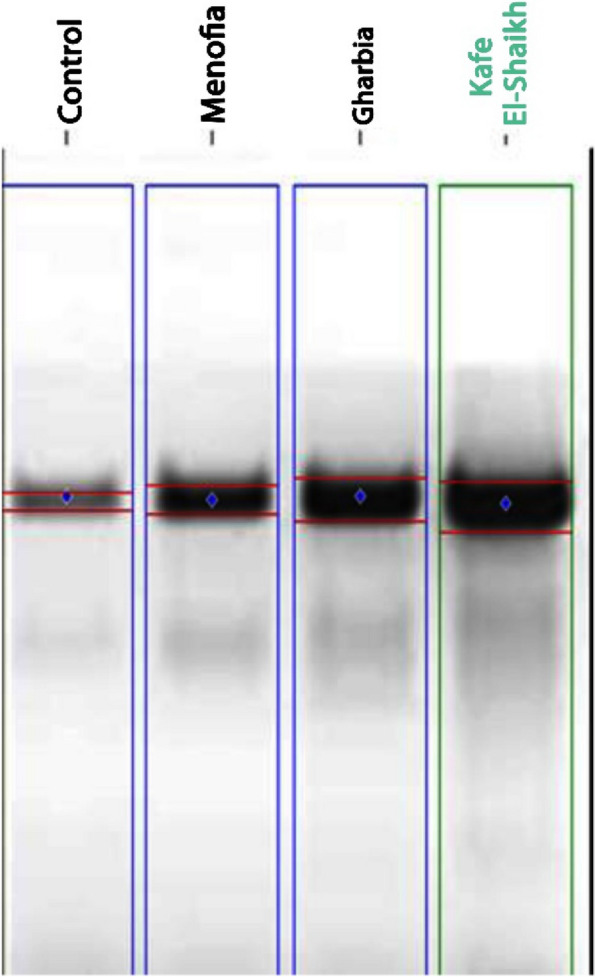
Metallothionein protein expression level for control and collected samples from El-Menofia, El-Gharbia and Kafr El-Sheikh locations

Metallothioneins, which consider the key role of protein to resist pollutant stresses, are significantly increased in all studied areas, compared to the control samples after exposure to heavy metals stresses (as showed Fig. 6 and Table 5). Increasing Metallothioneins intensity (15.4%) for collected Freshwater crayfish *Procambarus clarkii* from Kafr El-Shaikh location which remarked with high heavy metals concentrations area indicate its vital role for defense mechanism against heavy metals stresses, as the highest level of metalothioneins was observed in Kafr El-Shaikh (6.14), while the lowest was in El-Menofya (3.19), compared to the control group (1015).

**Table 5:** Expression level of Metallothionein signal for control and collected samples from El-Menofya, El-Gharbiya and Kafr El-Shaikh locations

Control	El-Menofya	El-Garbiya	Kafr El-Shaikh
Lane%	Lane%	Lane%	Lane%
1.15	3.19	4.74	6.14

**Fig. 7:**
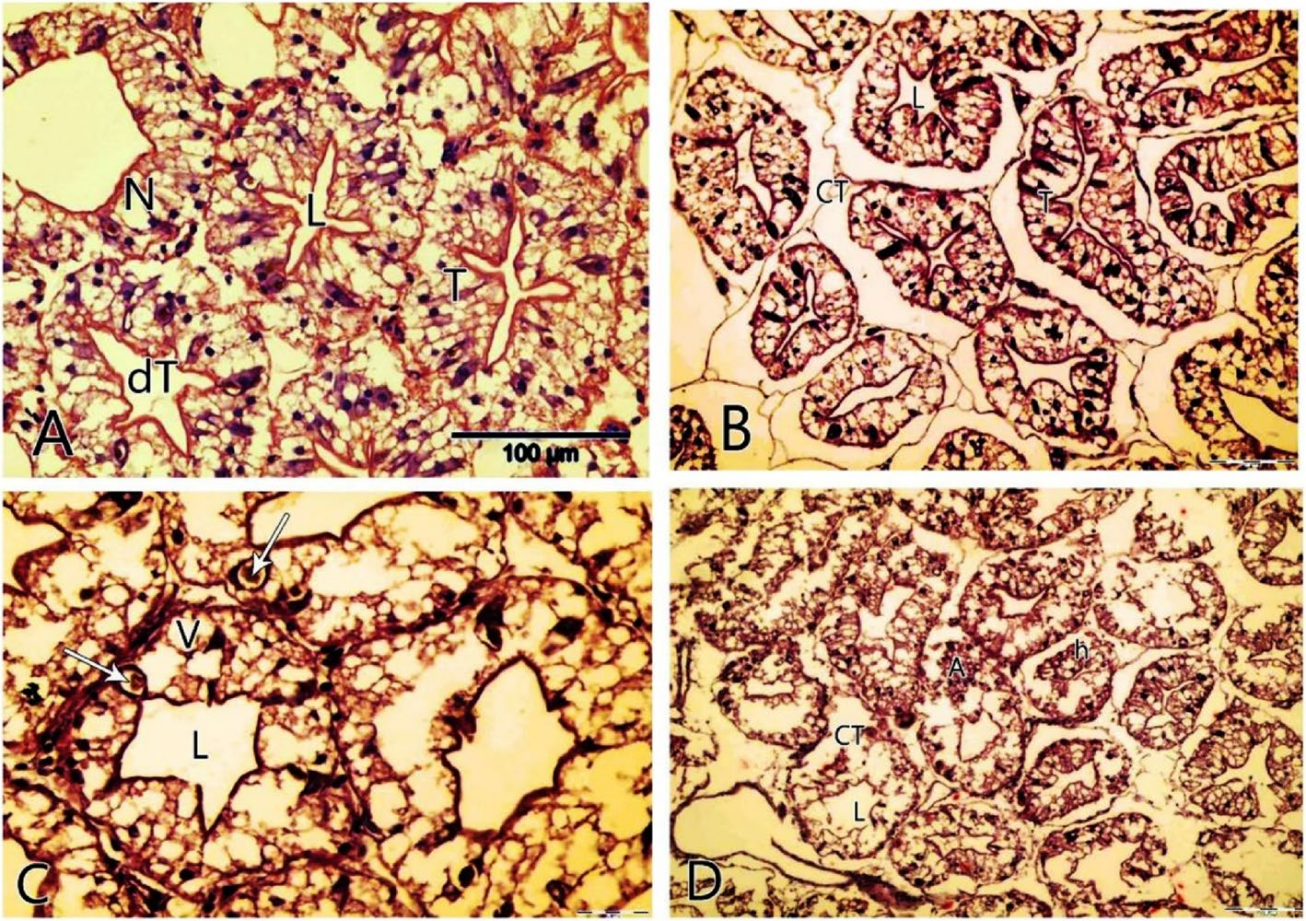
Photomicrograph of the cross-section of *P. clarkii* hepatopancreas stained with hematoxylin and eosin stain (H&E) showing: **A-** normal tissues in the control group. Tubules(T); lumen(l); Intertubular tissue(IT); and central, spherical nuclei(N). **B-** affected hepatopancreas tubules with heavy metals of collected *P. clarkii* from El-Gharbia showing irregular lumen (l); the basal lamina was rippled and connective tissue (CT); within the inter-tubular space. **C-** *P. clarkii* hepatopancreas from El-Monofia showed, that epithelial cells were eroded with vaculation and hemolytic infiltration was observed (Arrow). **D-** collected *P. clarkii* from Kafr- El- Sheikh showing atrophied epithelium(A); necrosis (n); lumen erosion and hyperplasia vaculation (V)

The histological observations on hepatopancreas dissected from different locations and control groups’ samples showed that the group of depurated specimens (control group) had normal hepatopancreas with hepatocytes and sinusoids which were arranged in lobular shape with an asterisk-like appearance lumen. Hepatocytes were polyhedral cells having central spherical nuclei; each lobule had an interlobular connective tissue (Fig. 7A). The effect of heavy metals in freshwater crayfish caused diffuse damage of the hepatopancreas which was characterized by necrosis involving the lobular shape with irregular lumen. In both El-Gharbiya and El-Menofya collected samples, the hepatopancreas displayed necrosis; vaculation and epithelial cells were eroded with vaculation and hemolytic infiltration was observed (Fig. 7B, C) respectively. However, hepatopancreas of collected samples collected from Kafr El-Shaikh showed the same symptoms with rupture epithelial necrosis with a proliferation of nucleolysis and lumen deformation (Fig. 7D).

## Discussions

Our findings in this manuscript was a continuation on results obtained in a previous research paper, in which heavy metals were measured in *Procambarus clarkii* tissues in the same studied areas [20]. Our results from this study directly indicated that heavy metals stressed on different oxidative stress markers including Superoxide dismutase (SOD), Catalase, and Glutathione S-transferase (GST) for collected Freshwater crayfish *P. clarkii* from three locations (Kafr El-Shaikh, El-Menofya, and El- Gharbiya). Changing oxidative stress markers of Freshwater crayfish Procambarus clarkii after exposure to heavy metals agreed with Zhang et al. [21] findings. They indicated significant changes in MDA content and antioxidant enzyme activity after Cd exposed *P. clarkii* to 2.0, 5.0, and 10.0 mg/L Cd for 24, 48, and 72 h. Also, our results came along with Kim et al. [22], who noticed an increase in SOD and GST activity after *Daphnia magna* exposure to Cd and Pb. In addition, Mi Kim et al. [23] results showed a significant increase in SOD and GST after exposure of copepod *Tigriopus japonicus* to Cd, Cu, and Zn.

In this study, different organs (Muscle, hepatopancreas, and gills) of collected Freshwater crayfish* P. clarkii* form three locations reflected varied responses against heavy metals stresses. Generally, gills showed the highest Superoxide dismutase (SOD), Catalase, and Glutathione S-transferase (GST) content compared with control gills, while hepatopancreas was the second highest organ in oxidative enzymes level. These results came side with Stara et al. [24] through recorded dramatic changes for antioxidant enzymes SOD and CAT, for hepatopancreas and muscle, of exposed Red Swamp Crayfish (*Procambarus clarkii*) to triazine herbicide. Also, our results came along with results of Wang et al. [25], who recorded a significant increase in SOD and GST activity after exposure of freshwater crab *Sinopotamon henanense*’s gills to Cd. Furthermore, an increase in SOD level in both gills and hepatopancreas of *Unio macus* was noticed after exposure to Cu [26]. The studies indicated that gill and hepatopancreas tissues are the most sensitive tissues exposed to toxicants. Since gills have a large surface in direct contact with water, they are considered a sensitive organ affected by water quality [27, 28].

It should be noticed that in our previous study [20], results showed that Kafr El-Shaikh governorate recorded the highest accumulation of all measured heavy metals (Cd, Cu, Fe, Pb, and Zn) in 2010, and also was the highest in accumulation of Cu and Pb in samples’ tissues, compared to other studied locations. These results definitely affected the oxidative enzymes measured in this study, as considering our heavy metals scanning findings, tissues of collected crayfish form distinguished Kafr El-Shiakh location were with the highest heavy metals content containing superior oxidative enzymes levels compared with collected tissues from El-Menofya and El-Gharbiya.

These findings agreed with Farombi et al. [29] findings that noticed a significant elevation in SOD activity after exposure to Cu in catfish *Clarias garepinus*. Also, Paris-Palacios et al. [30] recorded a significant increase in GST in the liver of *Rutilus rutilus* after Cu exposure.

As a co-factor for a number of enzymes, Cu^+2^ participates in oxidative stress and mitochondrial morphology with a narrow optimal range between essential and toxic concentrations. Numerous studies in a variety of species have shown that Cu^+2^ exposure resulted in apoptotic and autophagic cell death because of elevated ROS levels (Wang et al. [31]).

Monitoring and tracking protein profile and content for exposed Freshwater crayfish* P. clarkii* to increasing heavy metal stress levels revealed remarked variation in total protein content, band number, and polymorphism %.

Our results for a positive correlation between protein fractions and content of *P. clarkii* and exposure to increasing heavy metals were proved by Mohamed et al. [32]. They indicated that the total protein profile of *Artemia salina* tissues increased after treatment with Cd and Fe.

Cellular defense against metal toxicity is linked to the sequestration function of MT in the presence of toxic metals [33]. Heavy metals like zinc (Zn), copper (Cu), and cadmium (Cd) cause the promotion of MT in aquatic species when used in experiments [34]. These results have made MT a potentially useful biomarker for metal exposure. However, MT induction in aquatic species brought on by ambient exposure to heavy metals is more difficult to detect in field investigations, because environmental variables are less well characterized than in laboratory research [35]. Choosing a suitable organism to serve as a bioindicator is crucial [36].

Our results came along with Matin et al. [37], who found significant correlations between heavy metal levels and MT in hermit crab *Clibanarius signatus*. Similar results were showed by Hertika et al. [38], who stated that levels of MT among *Crassostrea iredalei* and *Crassostrea glomerata*, but always higher in gills than stomach. More support was added to our findings for increasing metallothioneins intensity after exposure to heavy metals by Viarengo and Nott [39], and Roesijadi [40]. They cleared that metallothioneins protein over expression in highly polluted sites may be related to its function for resistant pollutants via two main mechanisms. Firstly, preventing toxic potentials of non-essential metals through restriction of their binding to cells. Additionally, by providing a non-toxic zinc and copper pool for metalloenzyme production, the homeostasis of numerous cellular activities is enabled.

As mentioned before, Cu and Pb accumulation was the highest heavy metals in Kafr El-Shaikh governorate, compared to other locations. It was found that Cu is important for MT biosynthesis due to significant correlation more than Ni, Cr, Cd and Hg. A possible justification for the findings is related to that Cu is an essential trace metals and MT play a major role in its hemostasis in crabs [36]. Also, Cu was reported to promote MT synthesis in different tissues, such as digestive gland and gills of mussels [41]. In addition, the analysis of the relationship between MT values and heavy metals level in a very recent study, at 2023, showed the highly significant effect of lead concentration on MT concentrations on *Sulcospira testudinaria* [42].

Remarked variation for protein content of *P. clarkii* could be explained by heavy metal exposure in the light of Filipovic and Raspor’s [43] results. For lipoprotein cell repair, they described protein variation as a result of tissue organization, in particular cellular components of cytoplasmic cell membranes and organelles following exposure to pollutants.

Expressing new protein bands with low molecular weight (8 to 4.5 KDa) after exposure of *P. clarkii* to high heavy metal stresses in Kafr El-Sheikh location could be explained via Seebaugh and Wallace [44]. They fragmented metal-binding proteins (MTs) which stimulate Ubiquitin (Ub) /proteasome-dependent proteolytic systems that break down proteins for decreasing muscle mass which enables claw in land crab, *Gecarcinus lateralis*, and American lobster to the adapted withdrawal of the appendage at ecdysis. Moreover, Mohamed et al. [32] add more support for expressing new low molecular weight protein bands after exposure to heavy metals. They cleared that ubiquitin considers a low molecular weight protein (8.5 kDa) that is only expressed in *Artemia sp.* under stress, like inflammation or heavy metals exposure*.*

In comparison to the control, our findings revealed that protein polymorphism was present in every area of study. Kafr El-Shaikh governorate has the most polymorphism found. These findings corroborated those of David et al. [45], who discovered polymorphism of the MT gene as a result of the stress of summer mortality. Additionally, the Pacific oyster Crassostrea gigas was shown to have an MT polymorphism, which may be connected to its higher resistance to heavy metals [46].

Hepatopancreas is considered the primary organ for the generation of enzymes for metabolism, the contamination of heavy metals in the surrounding medium had a marked effect on the hepatopancreas leading to its damage [47]. The findings of this study demonstrated that the hepatopancreas of crayfish suffered substantial structural damage as a result of accumulating distribution levels. Non-essential elements have been shown in other investigations [26, 48] to accumulate in crayfish tissues as environmental concentration and exposure time increase.

## Conclusion

The results showed a clear effect of heavy metals accumulation on the Crayfish *P. clarkii* collected from three locations (Kafr El-Shaikh, El-Menofya, and El- Gharbiya), of the level of oxidative stress enzymes, and it also showed a remarked variation in total protein content, band number, and polymorphism %, in addition to a damaging effect on hepatopancreas compared to the depurated samples as control. The samples that were collected from the Kafr El-Shaikh area are considered the most affected by pollution, as the results showed the increasing Metallothioneins intensity (15.4%) which remarked with high heavy metals concentrations area.

These results indicated the vital role of MT as biomarker monitoring toxins that aquatic animals could be vulnerable to. Also, this work may highlights that accumulation of heavy metals could cause a genetic adaptation in some immune-related proteins as Metalothioneins, as a way of adaptation towards these contaminants.

## Material and methods

### Materials and methods

#### Sample collection

**Fig. 8:**
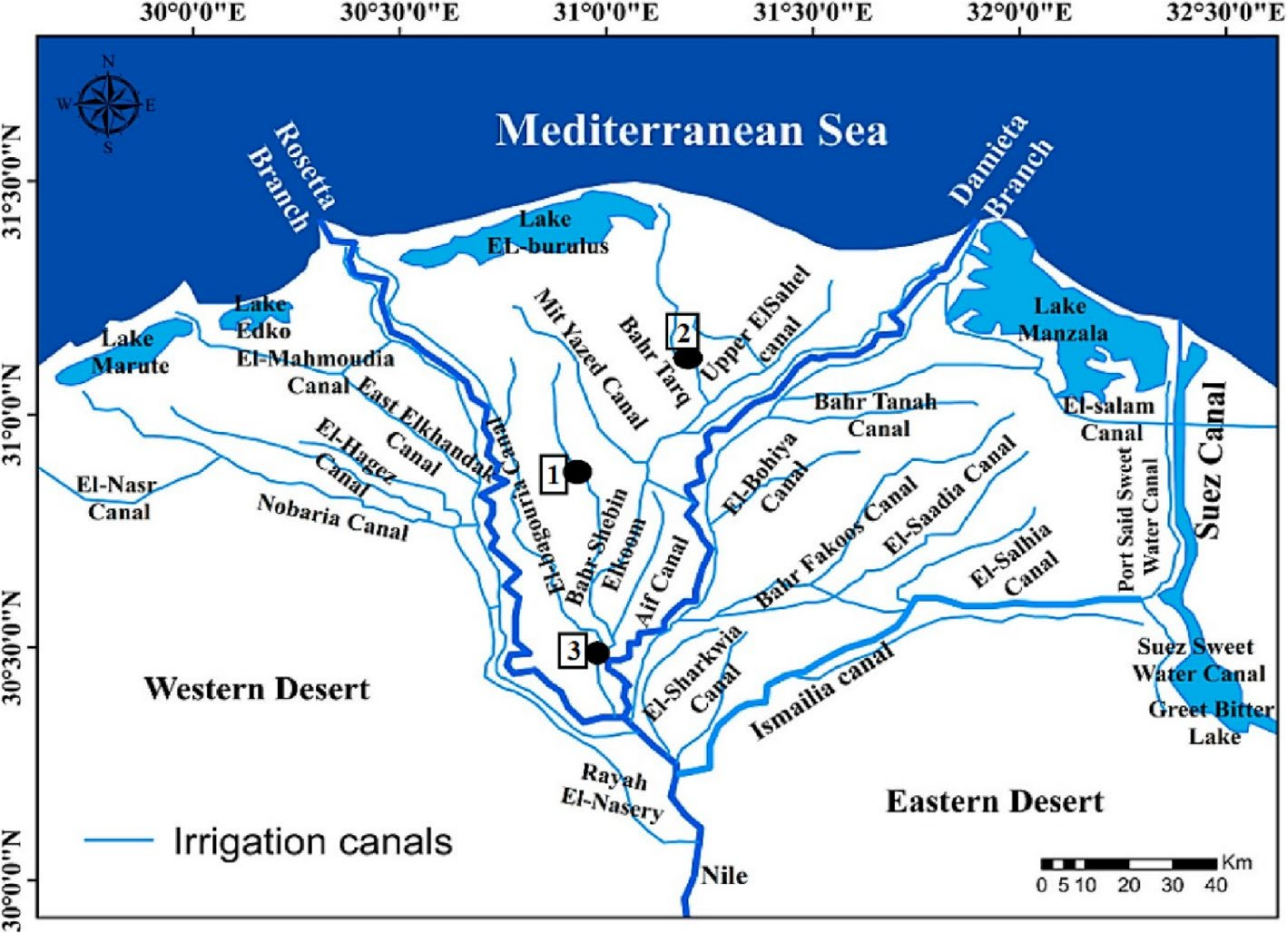
Map showing the three sampling locations in the Egyptian Nile Delta. 1, Gharbiya; 2, Kafr El-Shaikh; 3, Menofiya

Freshwater crayfish Procambarus clarkii samples were gathered in the spring from three Egyptian Delta^’^s governments: El-Gharbiya (30.824722°N30.815278°E), El-Menofya (30.52°N30.99°E), and Kafr El-Shaikh (31°06′42″N 30°56′45″E), as shown in Fig. 8 cited by Negm et al. [49].

Fifty samples from each area were collected, cleaned, and brought to a lab at the University of Tanta in plastic bags in an icebox.A group of Samples from each was directly dissected to obtain the Gills, abdominal muscles, and hepatopancreas then preserved (-20 ◦C) for enzymes and protein extraction. Another group of 40 samples was kept in the lab for three weeks (21 days) in glass tanks (*n* = 10/tank). The water was switched out every two days, and each tank was then filled with dechlorinated, well-aerated stored freshwater to a specific capacity. In the climate-controlled laboratory, an air conditioner was installed and set at 25 ^◦^C. The P. clarkii specimens underwent depuration for 21 days (Control group) before being dissected.

### Oxidative enzymes

Superoxide dismutase (SOD), Catalase (CAT), and Glutathione S-transferase (GST) estimation for Muscle, hepatopancreas, and Gills from Kafr El-Shaikh, El-Menofya, and El-Gharbiya locations, Superoxide Dismutase Activity Assay Kit (Colorimetric) (ab65354, Abcam), Catalase Activity Assay Kit (Colorimetric) (ab83464, Abcam) and GST Activity Assay Kit (Colorimetric) (ab65326) were used according to manufacturer protein.

### Protein extraction and purification methods

In this investigation, SDS Polyacrylamide gel electrophoresis (SDS-PAGE) with 12% T was applied as a biochemical marker to evaluate heavy metals toxicity for control and collected samples from Kafr El-Shaikh, El-Menofya, and El- Gharbiya locations according to the methods of Lammli [50].

TriFast (Peqlab, VWR company) (Isolation of RNA, DNA and Protein simultaneously).

#### Homogenization

In 1 ml TriFast, 50–100 mg of tissue or pellet was added. For efficient lysis use a glass- Teflon or power homogenizer. The sample volume should not exceed 10% of the volume of TriFast used for the homogenization.

#### Phase separation

RNA is forced exclusively into the aqueous phase whereas DNA and the proteins partition into the interphase and lower phenol phase. The volume of the RNA-containing phase is about 60% of the volume of the TriFast used for homogenization.

#### RNA precipitation

Precipitate the RNA with 0.5 ml of isopropanol per 1 ml of TriFast used for the initial homogenization. Keep samples on ice for 5–15 min and centrifuge for 10 min at 4 °C at 12,000 × g max. The RNA pellet should form a gel like precipitate on the bottom and on the side of the tube.

#### DNA precipitation

DNA is sedimented by centrifugation at 2,000 × g at 4 °C for 15 min. (Careful removal of the aqueous phase is critical for the quality of the isolated DNA).

#### Protein precipitation

Precipitate the proteins from the ethanol/phenol supernatant with 1.5 ml Isopropanol. Keep the samples in washing solution for 20 min at room temperature before centrifuging at 7,500 × g for 5 min at 4 °C. Next vortex the protein pellet once with 2 ml of 100% ethanol, store for 20 min at room temperature and centrifuge at 7,500 × g for 5 min at 4 °C.

#### Protein solubilization

Remove any ethanol and dry the Protein pellet briefly for 5–10 min under vacuum and dissolve it in 1% SDS by pipetting it up and down. Incubation of the samples at higher temperatures (50–100 °C) may be necessary to yield complete solubilization.

### Protein fraction quantification

Based on the interaction of the protein with Coomassie Brilliant Bleu G250 (CBBG-250) under acidic conditions, the protein fraction concentration was calculated using Bradford's [51] technique. According to Bamdad et al. [52], 12% slab gel was used.

After gel polymerization, 30 g of proteins were added to each sample, and electrophoresis was run at 75 V through a stacking gel for roughly 2 h.

### Metallothionein (MT) Protein expression

GE Healthcare's HybondTM nylon membrane was used to electrophorese proteins on SDS-PAGE. The membrane was then treated overnight at 4°C with an antibody solution comprising the primary antibody Anti-Anti Metallothionein (Abcam, ab192385). Also, Anti- β-actin (Abcam, ab8226) was used as a housekeeping gene [53–55].

- Membrane was washed at room temperature for 30–60 min with 5 or more changes of Blotting Buffer.
- Membrane was incubated for 1 h at room temperature in an Antibody Solution containing appropriate dilution of HRP-conjugated secondary antibody (Antibody concentration. 0.1–0.5 µg/mL. Adjust antibody concentration from 0.05 to 2.0 µg/mL to obtain desired signal strength and low background.
- Membrane was washed for 30–60 min with 5 or more changes of Blotting Buffer.

### Light microscopic preparations

Dissected Hepatopancreas of the collected and control (14 days depurated samples) Samples were preserved in 10% formalin and dehydrated via an ascending series of ethanol, embedded in paraffin wax, sectioned at 4–6 μm thick, and stained with hematoxylin and eosin. An Olympus (E330-ADU1.2X) microscope with a digital camera was used to take photomicrographs Image-Pro Express software (Media Cybernetics).

### Data analysis

Statistical analysis was performed by SPSS version 9.0 software. The enzymes differences between the control and the locations groups were analyzed by one-way analysis of variance (ANOVA). A value of *P* < 0.05 was accepted as statically significant. All data are expressed as the mean ± standard deviation (SD). Gel documentation system (Geldoc-it, UVP, England), was applied for data analysis using Totallab analysis software, ww.totallab.com, (Ver.1.0.1).

## Data Availability

The datasets used and analyzed during the current study available from the corresponding author on reasonable request.

## Abbreviations

Ag: Chemical symbol for Silver
AHD: Aswan High Dam
ANOVA: One way analysis of variance
BSA: Bovine Serum Albumin
CAT: Catalyse
Cd: Chemical symbol for Cadmium
Cr: Chemical symbol for Chromium
Cu: Chemical symbol for Copper
DNA: Deoxyribonucleic acid
Fe: Chemical symbol for Iron
GPX: Glutathione peroxidase
GSH: Glutathione
GSTs: Glutathione S-Transferases
H_2_O_2_: Hydrogen peroxide
Hepato: Hepatopancreas
Hg: Chemical symbol for Mercury
IACUC: Institutional Animal Care and Use Committee
KDa: KiloDalton
MTs: Metallothioneins
n: Number
Ni: Chemical symbol for Nickel
OD: Optical Denisty
P. clarkii: Procambarus clarkia
Pb: Chemical symbol for Lead
REC: Research ethical Committee
RNA: Ribonucleic acid
ROS: Reactive Oxygen Species
SD: Standard Deviation
SDS: Sodium dodecyl-sulfate polyacrylamide gel electrophoresis
SOD: Super Oxide Dismutase
SPSS: Statistical Package for the Social Sciences
Zn: Chemical symbol for Zinc
